# Dengue Viral RNA Levels in Peripheral Blood Mononuclear Cells Are Associated with Disease Severity and Preexisting Dengue Immune Status

**DOI:** 10.1371/journal.pone.0051335

**Published:** 2012-12-19

**Authors:** Anon Srikiatkhachorn, Sineewanlaya Wichit, Robert V. Gibbons, Sharone Green, Daniel H. Libraty, Timothy P. Endy, Francis A. Ennis, Siripen Kalayanarooj, Alan L. Rothman

**Affiliations:** 1 Department of Medicine, University of Massachusetts Medical School, Worcester, Massachusetts, United States of America; 2 Department of Virology, Armed Forces Research Institute of Medical Sciences, Bangkok, Thailand; 3 Department of Medicine, State University of New York, Upstate Medical University, Syracuse, New York, United States of America; 4 Queen Sirikit National Institute of Child Health, Bangkok, Thailand; 5 Institute for Immunology and Informatics, University of Rhode Island, Providence, Rhode Island, United States of America; Oxford University, Viet Nam

## Abstract

**Background:**

Infection with dengue viruses (DENV) causes a wide range of manifestations from asymptomatic infection to a febrile illness called dengue fever (DF), to dengue hemorrhagic fever (DHF). The in vivo targets of DENV and the relation between the viral burden in these cells and disease severity are not known.

**Method:**

The levels of positive and negative strand viral RNA in peripheral blood monocytes, T/NK cells, and B cells and in plasma of DF and DHF cases were measured by quantitative RT-PCR.

**Results:**

Positive strand viral RNA was detected in monocytes, T/NK cells and B cells with the highest amounts found in B cells. Viral RNA levels in CD14+ cells and plasma were significantly higher in DHF compared to DF, and in cases with a secondary infection compared to those undergoing a primary infection. The distribution of viral RNA among cell subpopulations was similar in DF and DHF cases. Small amounts of negative strand RNA were found in a few cases only. The severity of plasma leakage correlated with viral RNA levels in plasma and in CD14+ cells.

**Conclusions:**

B cells were the principal cells containing DENV RNA in peripheral blood, but overall there was little active DENV RNA replication detectable in peripheral blood mononuclear cells (PBMC). Secondary infection and DHF were associated with higher viral burden in PBMC populations, especially CD14+ monocytes, suggesting that viral infection of these cells may be involved in disease pathogenesis.

## Introduction

Infection with dengue viruses (DENV) causes a wide range of manifestations from asymptomatic infection to a febrile illness called dengue fever (DF), to dengue hemorrhagic fever (DHF), a viral hemorrhagic disease characterized by plasma leakage and bleeding diathesis. [1] The mechanisms of severe dengue disease are not completely elucidated. Both higher viral burden and enhanced immune activation have been associated with severity. [2] An important risk factor is a secondary DENV infection in a host previously infected with DENV of another serotype. Cross-reactive, non-neutralizing DENV-specific antibodies have been shown to enhance viral attachment and uptake by immunoglobulin receptor-bearing target cells, which may lead to higher viral loads and more intense immune activation. [3], [4]

The in vivo cellular targets of DENV remain to be completely identified. Studies of human dengue cases have identified macrophages and lymphoid cells as major targets of DENV as supported by the presence of viral antigen and RNA. [5]–[9] However, the relation of viral burden in various cell populations to clinical severity and host immunological status has not been examined. We measured DENV RNA in major cellular constituents of peripheral blood mononuclear cells (PBMC) in dengue cases and identified B cells as the principal cells harboring DENV in both DF and DHF cases. Secondary infection and DHF were associated with higher viral burden in selective populations of PBMC.

## Materials and Methods

### Ethics Statement

Written informed consent was obtained from the parent or the legal guardian of each study subject. The study was approved by the Institutional Review Boards of the Thai Ministry of Public Health, the US Army Surgeon General's Office and the University of Massachusetts Medical School.

### Clinical study design

The study design has been previously described. [10] Children 6 months to 18 years of age who presented with febrile illness without an obvious source of infection within 72 hours after fever onset at Queen Sirikit National Institute of Child Health were enrolled and observed in hospital until one day after defervescence and until clinically stable. Clinical and laboratory data including hematocrit, platelet counts, liver enzyme (AST, ALT), and albumin levels were collected daily. Plasma and PBMC samples were collected daily during hospitalization and at a follow-up visit during convalescence (four to six days after discharge). Plasma were divided into aliquots and stored at −70°C. DENV infection was confirmed by serology and by viral isolation and/or RT-PCR of plasma. [11], [12] Confirmed dengue cases were classified as primary or secondary infection on the basis of IgM/IgG ELISA as previously described. [12] Subjects were classified as DF or DHF according to the World Health Organization criteria. [1] The day of defervescence was defined as fever day 0, and the days before (fever days −1, −2, etc.) and after (fever days +1, +2, etc.) were numbered sequentially. All patients had a right lateral decubitus chest radiograph performed one day after defervescence. The size of the effusion was expressed as pleural effusion index (PEI) = the vertical dimension of the fluid/the width of the hemithorax ×100. Hemoconcentration was defined as the percentage difference between peak hematocrit reading during acute illness and the baseline hematocrit reading at early convalescence.

### Sample Selection

Samples were selected from archived specimens of clinically and serologically characterized cases. Samples were selected to include a representative subset of subjects from the parent study with sufficient specimens available for isolation of PBMC subsets. To provide data on changes over time in DENV RNA levels, we limited our selection to cases with samples available from multiple (at least 2) days during the febrile period. Samples from DF and DHF cases, cases with primary and secondary DENV infection, and viral serotypes were randomly selected. Twenty cases of DF and fifteen cases of DHF were selected (Table 1). The clinical, demographic, and virologic characteristics of the selected cases and of all dengue cases enrolled during the same period are shown (Table S1). Except the proportions of viral serotypes, there were no statistically significant differences in the characteristics of the DF and DHF cases selected for the study and all cases enrolled during the same period. To compare the viral RNA levels in PBMC subpopulations during primary and secondary infections, we performed quantitative RT-PCR of cells collected on fever day 2 from 11 primary (4 cases as indicated in table 1 and an additional 7 cases) and 15 secondary DENV infections. The demographic and laboratory characteristics of these cases are shown (Table S2).

**Table 1:** Patient characteristics.

	Clinical Diagnosis	
	DF	DHF
**Number of cases**	20	15
**Sex (M/F)**	13/7	9/6
**Age (mean(SD))**	8.7 (2.5)	9.3 (3.4)
**Dengue serotypes**		
**DENV1**	5	4
**DENV2**	5	5
**DENV3**	6	3
**DENV4**	4	3
**Serology**		
**Primary infection**	4	1
**Secondary infection**	16	14
**Hemoconcentration (%)** * **(mean(SD))**	9.3 (7.8)	15.75 (12)
**Lowest platelet counts** * **(mean(SD)**	110350 (44909)	54066 (31605)
**Peak AST (u/dl) (mean(SD)**	107 (94)	160 (111)
**Peak ALT (u/dl) (mean(SD)**	57 (42)	95 (103)
**Lowest albumin (g/dl)** * **(mean(SD)**	4.15 (.49)	3.88 (.57)
**Pleural effusion index (%) (mean (range))**	0	17.4 (0–56.6)

* Indicates statistically significant difference between DF and DHF (P<.05). Two DHF cases met the definition of severe dengue according to the 2009 clinical classification system.

### PBMC isolation, storage, and fractionation

PBMC were isolated from whole blood by Ficoll-Hypaque density gradient procedure. Cells were washed twice with RPMI and resuspended in freezing media containing 20% fetal calf serum, 10% DMSO in RPMI at 1–2×10^6^cells/ml. Cells were gradually cooled at −70°C for 18 hours and then stored in the vapor phase of liquid nitrogen for long term storage. One aliquot containing 1–2×10^6^ cells was preserved in RNA preserving solution and stored at −70°C. [13] To obtain subpopulations of PBMC, cells were thawed and fractionated by sequential positive selection using antibody-conjugated magnetic beads into CD14+, CD2+, and CD20+ cells (Milteyni, Auburn, CA). The purity of the fractionated cells was evaluated by immunostaining for cell surface markers and flow cytometry.

### Quantitative RT-PCR

Fractionated cells were lysed in Trizol solution (Life Technologies, Grand Island, NY) and RNA was extracted according the manufacturer's instructions. RNA was also extracted from 100 µL of plasma. Reverse transcription and PCR for positive strand DENV RNA were performed as described. [2] Primers used in RT-PCR are shown in Table S3. Negative strand DENV RNA was quantitated using tagged RT-PCR, a technique shown to avoid false priming and thereby provide greater strand-specific detection. [14] Briefly, a primer (TagF) which anneals to a sequence common in all four dengue viruses and contains an additional 5′ exogenous sequence was used for reverse transcription. The cDNA samples were purified with column to remove excess primers. Quantitative PCR was performed with an upstream primer containing only the tag sequence and serotype specific downstream primers for DENV-1,- 2, and -3, and for DENV-4. The amplified products of both the positive and negative strand-derived cDNA were detected using the same set of fluorogenic probes as both reactions amplified nearly identical segments of the cDNA. The sample viral RNA concentrations (copies/ml) were estimated based on a standard curve generated with known concentrations of plasmid DNA containing dengue viral sequence.

The number of cells in each PCR reaction was estimated by quantitative RT-PCR for β2-microglobulin mRNA using primers- B2M-forward (5′TGCTCGCGCTACTCTCTCT3′) and B2M-reverse (5′TCCATTCTCTGCTGGATGAC3′) in RT-PCR containing Sybr green (Biorad, Hercules, CA). The cell numbers were estimated based on a standard curve generated with known numbers of PBMC. Cell-associated DENV RNA levels were expressed as copies/million cells following the formula: copies/million cells = ((viral RNA concentration (copies/ml)×PCR volume (ml))/cells per PCR)×10^6^. All samples were assayed in duplicate and the mean values were used for analyses. The ABI gene detection system 7700 (Perkin-Elmer Applied Biosystems, Carlsbad, CA) was used for PCR cycling amplification, data collection, and analysis.

### Statistical Analysis

Continuous variables were compared between groups using Mann Whitney Test. Wilcoxon signed rank Test was used to analyze differences in variables of individuals at different time points or related variables within individual samples. Categorical variables were compared between groups using Chi square test. Correlations were analyzed using Spearman method. Multivariate non-parametric test of independence was performed with Pillai's trace test. A *P* value<0.05 was considered to indicate statistical significance. All tests were two sided.

## Results

To assess the viral burden in plasma and various populations of PBMC during DENV infection, we performed quantitative RT-PCR for DENV RNA in 20 DF cases and 15 DHF cases (Table 1). All of the subjects selected had specimens available from fever days −2 and −1. These cases represented approximately 10% of all dengue cases enrolled during the same period and were not significantly different in age, sex and serological status from the whole cohort (Table S1). The notable difference is the relative proportion of virus serotypes of the studied cases compared to that of the whole cohort. This was due to our study design to include roughly equal numbers of cases with each DENV serotype for this analysis. The DF and DHF cases were not different in demographic or serological characteristics. As expected, the mean nadir platelet counts and albumin levels were lower in DHF cases (Table 1). Two DHF cases with narrowed pulse pressure met the definition of severe dengue according to the 2009 clinical classification scheme. [15]

**Figure 1:**
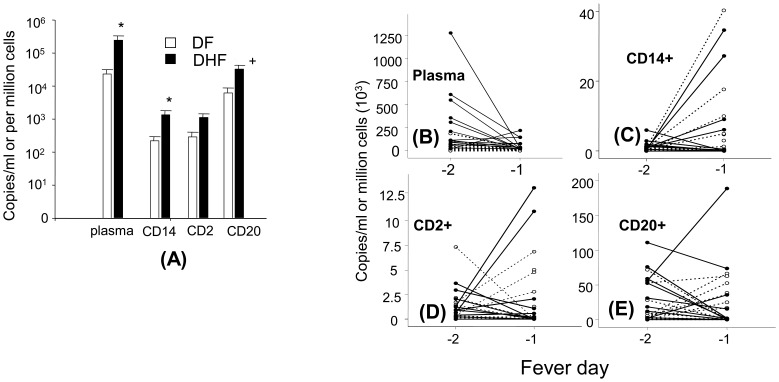
Dengue virus in plasma and peripheral blood mononuclear cells. Comparisons of dengue viral (DENV) RNA levels in plasma and PBMC subpopulations in DF and DHF cases two days prior to defervescence (fever day −2) (A). * indicates statistically significant differences in DENV RNA levels between DF and DHF (P<.05). +indicates differences between DENV RNA levels in CD20+ cells compared to CD14+ and CD2+ cells (P<.05). (B, C, D, E) Levels of DENV RNA levels in plasma (B), CD14+ (C), CD2+ (D) and CD20+ (E) cells on fever day −2 and fever day −1. Each line represents individual cases: solid lines: DHF, dashed lines: DF.

**Figure 2:**
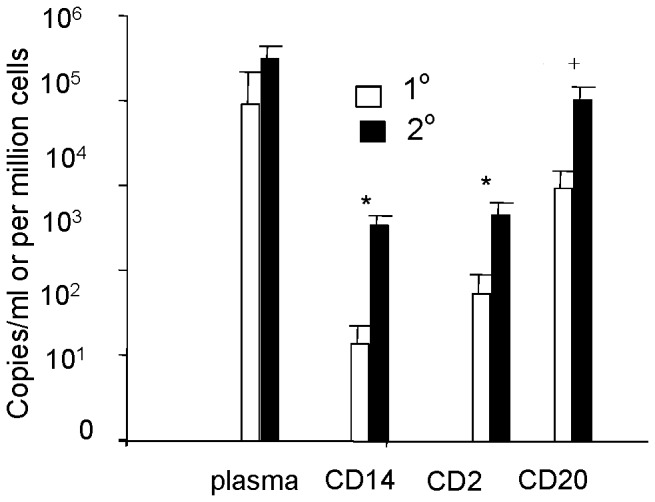
DENV RNA levels in plasma and PBMC subpopulations in primary and secondary infections. Plasma and PBMC cell subpopulations of 11 primary and 15 secondary DENV infections, all of whom had DF, were analyzed. * indicates statistically significant differences in DENV RNA levels between primary and secondary infection (P<.05). +indicates differences between DENV RNA levels in CD20+ cells compared to CD14+ and CD2+ cells (P<.05).

On the basis of previous studies indicating that viremia declines at the time of defervescence [2] we selected plasma and PBMC samples collected prior to defervescence for this analysis. PBMCs were fractionated into CD14+ (monocytes), CD2+ (T and NK cells), and CD20+ (B cells) cell fractions. The purity of each fraction exceeded 90%, as assessed by flow cytometry. Quantitative RT-PCR of fractionated cell RNA detected DENV RNA in all fractions (Figure 1), with CD20+ cells harboring higher viral RNA levels compared to CD14+ cells and CD2+ cells. The distribution of DENV RNA among cell populations was similar in DF and DHF. Plasma DENV RNA levels were significantly higher in DHF cases compared to DF cases (p = .001) (Figure 1A). The CD14+ cell fractions from DHF cases also contained higher DENV RNA levels than those from DF cases (p = .01). Plasma viral RNA levels declined significantly from fever day −2 to fever day −1 in all cases (p = .001) (Figure 1B). The viral RNA in cell fractions did not change significantly between these 2 time points) (Figure 1 C, D, E). To examine the effects of host serological status on cellular viral burden, we compared the levels of DENV RNA in PBMC subpopulations of 11 primary and 15 secondary DENV infections, all of whom had DF. As shown in figure 2, viral RNA levels in CD14+ and CD2+ cell fractions were higher in patients with secondary infection than those with primary infection. There was no significant difference in plasma viral RNA levels between primary and secondary infections.

To determine whether there was evidence of active DENV replication in PBMC, we performed strand-specific quantitative RT-PCR to detect negative strand DENV RNA using tagged RT-PCR. [14] Experiments employing this technique to measure the amount of positive and negative viral RNA in DENV-infected C6/36 cells revealed that the negative strand RNA levels were 1–2% of positive strand RNA (Table S4). No negative strand RNA was detected in RNA extracted from uninfected cells or when the Tag sequence primer was omitted from the PCR of DENV infected cells.

**Figure 3:**
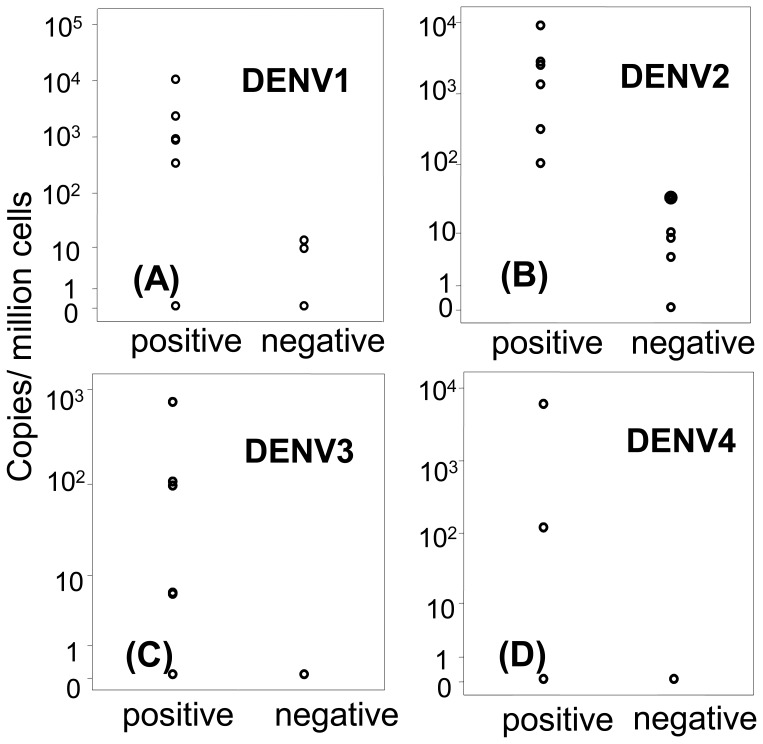
Positive and negative strand DENV RNA levels in unfractionated PBMC. Quantitative PCR for positive and negative strand viral RNA was performed with RNA extracted from unfractionated PBMC collected on fever day-2 from subjects infected with DENV1 (A), DENV2 (B), DENV3 (C), or DENV4 (D). The range of the positive strand DENV RNA was 0 to 6069 copies/million cells. Negative strand DENV RNA was detected in 6 of 18 samples with detectable positive strand RNA and the levels ranged from 5 to 35 copies/million cells.

We performed quantitative RT-PCR for both positive and negative strand genomes using RNA samples extracted from PBMC preserved in RNA preserving solution at the time of collection (fever day −2). As shown in figure 3, we detected very low copy numbers of negative strand DENV RNA in a few of these PBMC samples, indicating that very little active viral replication is detectable in PBMC.

We examined the correlations between viral RNA levels and parameters of disease severity. The levels of viral RNA in plasma and CD14+ cells on fever day-2, but not in CD2+ or CD20+ cells, correlated with the size of pleural effusions, an indicator of the severity of plasma leakage (Table 2). In agreement with this, nadir albumin levels inversely correlated only with viral RNA levels in plasma and CD14+ cells. Although the levels of viral RNA in plasma correlated significantly with RNA levels in CD14+ cell fraction (r^2^ = 0.474, P = .001), multivariate analysis showed that these parameters independently correlated with pleural effusion index (P = .001). Nadir platelet counts showed an inverse correlation with viral RNA levels in CD2+ cells. Viral RNA levels in plasma or cell fractions did not correlate with hemoconcentration or hepatic transaminase levels.

**Table 2:** Univariate correlations between indicators of disease severity and viral RNA levels in plasma and cell fractions collected on fever day-2.

Source of viral RNA	Correlations with indicators of disease severity (R (p value))					
	Pleural effusion index	Nadir albumin levels	Liver enzyme (AST)	Liver enzyme (ALT)	Hemo concentration	Nadir platelet counts
**Plasma**	**.532 (.002)**	**−.466 (.008)**	.015 (.934)	.15 (.397)	.103 (.562)	−.209 (.252)
**CD14+**	**.477 (.008)**	**−.466 (.033)**	.115 (.543)	.15 (.425)	.259 (.166)	−.430 (.05)
**CD2+**	.29 (.114)	−.265 (.157)	.175 (.346)	.277 (.131)	.237 (.199)	**−.479 (.038)**
**CD20+**	.353 (.098)	−.071 (.747)	.185 (.399)	.142 (.519)	.223 (.307)	−333 (.141)

Correlations between viral RNA levels in plasma and cell fractions on fever day −2 and indicators of disease severity were analyzed by Spearman's Rho test. Multivariate non-parametric test of independence revealed that plasma viral RNA levels and viral RNA levels in CD14+ cell fraction independently correlated with pleural effusion index (P = .001).

## Discussion

Our results demonstrate that peripheral blood B cells, T cells, and monocytes harbor DENV genomes during acute infection. The DENV RNA content associated with each of these cell populations was low (less than 1 copy/cell), however, indicating that no more than a small minority of cells within each fraction contains DENV in vivo. Consistent with this finding, we have been unable to detect DENV infected peripheral blood cells by flow cytometry (unpublished observation). Our results are in agreement with a previous study demonstrating low viral detection and recovery rate from PBMC. [16] This is in contrast to flow cytometric studies demonstrating high frequencies of peripheral blood cells, including B cells and monocytes, expressing DENV antigens [7], [8]. Although antibodies to DENV structural proteins could detect cells that have taken up viral particles without productive infection, one study using an antibody specific to the DENV NS3 protein suggested that active viral replication was occurring in ∼10% of CD14+ cells. [8] However, the strikingly high percentages of DENV-infected cells in peripheral blood reported in these studies were not corroborated by a study employing in situ hybridization. [6] Differences in study design and populations may explain these disparate results; further studies are needed to provide clarification.

We found that B cells harbored the highest viral RNA levels among the cell types studied. This is consistent with other studies identifying B cells as the major cell population in peripheral blood that expressed DENV antigens and harbored infectious virus. [7], [9] In addition, an in situ hybridization study of spleen and lymph nodes of fatal dengue cases revealed DENV RNA in what appeared to be centroblasts in germinal centers, indicating the presence of DENV in B cells in secondary lymphoid organs. [6] Since circulating B cells are in transit from secondary lymphoid tissues to bone marrow [17], it is possible that antigenic stimulation in secondary lymphoid tissue leads to the expansion of B cells expressing DENV-specific antibodies, which facilitate DENV capture and entry into these cells. A recent study demonstrating high frequencies of dengue-specific plasmablasts in peripheral blood during an acute infection supports this notion. [18] The detection of viral RNA in CD2+ cells was somewhat unexpected. Although activated T cells have been shown to support DENV replication in vitro [19], to our knowledge this is the first documentation of DENV RNA in T/NK cells in clinical samples.

High plasma viral load has been associated with increased severity of dengue illness. [2] We have demonstrated in this study that, during secondary infections, viral load in CD14+ cells is also higher in DHF compared to DF. Conversely, secondary DENV infection was also associated with higher viral RNA levels in CD14+ and CD2+ cell fractions. The higher cell-associated DENV RNA in secondary infection may be mediated by cross-reactive antibodies that enhance viral capture and entry into peripheral blood cells via Fc receptors. The relative abundance of dengue-specific IgG and IgM antibodies and the expression of various Fc receptors for IgG and IgM by different cells may determine the cellular distribution of viral RNA during primary and secondary infections. However, we did not observe differences in the cellular distribution of viral RNA between primary and secondary infections. It is possible that differences may exist between subpopulations not delineated in this study such as activated T and B cells.

Studies have demonstrated that monocytes, B cells, and activated T cells can support DENV replication in vitro. [19], [20] However, a study employing in situ hybridization with sense ribonucleotide probes failed to identify replicative intermediate of DENV in human tissue samples. [6] Using a negative strand-specific PCR technique, we found negative strand DENV RNA only in a few PBMC samples and the levels were <1% of the positive strand DENV RNA levels in the same sample. This suggests that viral replication is occurring in a minority of PBMC that harbor viral RNA. Another study employing quantitative RT-PCR reported up to 10,000 copies of negative strand DENV RNA per million PBMC, which represented 1–5% of positive strand DENV RNA genome copies and was interpreted to indicate active DENV replication. [21] However, the amount of negative strand DENV RNA detected in that study might have been overestimated, as a tagged primer technique was not used.

We observed a decline in plasma DENV RNA levels at the time of defervescence as previously reported. [2] In contrast, no contemporaneous decline in cell-associated virus was observed. The sustained viral burden in PBMC might be relevant in dengue pathogenesis since DENV-infected cells serve both as producers of pro-inflammatory cytokines, including TNFα and IL-6 [20] and also as antigen-presenting cells capable of inducing cytokine production by DENV-specific T cells. We did not note any association between the kinetics of cell-associated DENV RNA and disease severity. Our study was limited to peripheral blood, however, and may not fully reflect the kinetics of DENV RNA in tissues.

Our study demonstrated several correlations between indicators of disease severity and viral RNA levels in plasma or PBMC populations. Interestingly, indicators of plasma leakage (pleural effusion index and nadir albumin levels) showed statistically significant associations only with viral RNA levels in plasma and CD14+ cells. The selective correlation of plasma leakage with viral RNA levels in CD14+ cells suggests that these cells may play an important and direct role in this disease process. DENV-infected monocytes/macrophages have been shown to produce proinflammatory cytokines with permeability-enhancing effects. [20], [22] Notably, there was a significant correlation (r^2^ = 0.474) between viral RNA levels in plasma and in CD14+ cells. Therefore, the relative contributions of virus in these two compartments to plasma leakage could not be clearly delineated. It is also possible that the viral RNA levels in these two compartments are mechanistically related and both may contribute to plasma leakage. Similar correlations were not observed between viral RNA levels and hemoconcentration. However, hemoconcentration can be obscured by hemorrhage and fluid treatment, both of which are more common in DHF cases. We also observed an association between nadir platelet counts and CD2+ cell viral load. CD2+ T cells including NK and T cells may be activated by virus-antibody complexes and mediate antibody-dependent cytolysis directed against platelet or platelet progenitors expressing viral antigens on their surface.

Our findings are subject to several limitations. The number of cells required for cell sorting and quantitative RT-PCR analysis limited the number of cases that could be studied. However, the clinical and demographic characteristics of the selected cases did not significantly differ from those of the total dengue cases enrolled during the same period, suggesting that we did not introduce any bias by our selection. The paucity of DHF cases with a primary infection precluded the analysis of a relation between clinical severity and viral RNA levels in primary infections. The small number of samples representing each viral serotype in this study might have limited our ability to detect serotype-associated differences in DENV RNA distribution among cell populations. In addition, our assay method would not differentiate between surface and intracellular virus, a distinction that may have implications on cell signaling and cytokine responses. Studies have shown that the interaction of virus-antibody complexes with Fc receptors may induce the production of inhibitory cytokines, such as IL-10. [23], [24] On the other hand, DENV infection has been shown to induce proinflammatory cytokines including chemokines, TNF-α, and interferons. [20], [22], [25] Since archived frozen cell samples were used in this study, our analysis might have included only cells that survived cryopreservation but not cells that might have undergone activation induced cell death particularly T cells. Further studies that examine fresh samples from cases with different viral serotypes and employ techniques that can differentiate between viral infection and surface viral attachment will be needed to answer these questions.

## Supporting Information

Table S1 Patient characteristics of the all dengue cases enrolled between 1994–2001 and dengue cases selected for this study.
(DOCX)

Table S2 Patient characteristics of dengue fever (DF) cases with either primary or secondary infection.
(DOCX)

Table S3 Primers used in positive and negative strand RT-PCR.
(DOCX)

Table S4 DENV RNA in C6/36 cells detected by positive and negative strand PCR.
(DOCX)
