# Supplementary material for: Dengue Viral RNA Levels in Peripheral Blood Mononuclear Cells Are Associated with Disease Severity and Preexisting Dengue Immune Status
Source: PLoS One. 2012 Dec 19;7(12):e51335. doi: 10.1371/journal.pone.0051335 (PMC3526575; doi:10.1371/journal.pone.0051335)
Supplement: Table S1 — Patient characteristics of the all dengue cases enrolled between 1994–2001 and dengue cases selected for this study. (DOCX) [file pone.0051335.s001.docx]

**Supplemental table S1** Patient characteristics of the all dengue cases enrolled between 1994-

|  | **Clinical Diagnosis** | |
| --- | --- | --- |
|  | **DF** | **DHF** |
| **Number of cases** |  |  |
| **All cases** | 189 | 119 |
| **Selected cases** | 20 | 15 |
| **Sex (M/F)** |  |  |
| **All cases** | 99/90 | 69/50 |
| **Selected cases** | 13/7 | 9/6 |
| **Age (mean(SD))** |  |  |
| **All cases** | 7.8 (2.9) | 8.3 (3.1) |
| **Selected cases** | 8.7 (2.5) | 9.3 (3.4) |
| **Dengue serotypes**  **DENV1/ DENV2/ DENV3/DENV4** |  |  |
| **All cases** | 54/36/55/5* | 26/27/30/2 |
| **Selected cases** | 5/5/6/4* | 4/5/4/2 |
| **Serology**  **(primary/secondary)** |  |  |
| **All cases** | 70/119 | 13/106 |
| **Selected cases** | 4/16 | 1/14 |

2001 and dengue cases selected for this study.

* Indicates statistically significant difference between selected cases and all enrolled cases by Chi’s square test (P = .018)
