# Supplementary material for: Dengue Viral RNA Levels in Peripheral Blood Mononuclear Cells Are Associated with Disease Severity and Preexisting Dengue Immune Status
Source: PLoS One. 2012 Dec 19;7(12):e51335. doi: 10.1371/journal.pone.0051335 (PMC3526575; doi:10.1371/journal.pone.0051335)
Supplement: Table S2 — Patient characteristics of dengue fever (DF) cases with either primary or secondary infection. (DOCX) [file pone.0051335.s002.docx]

**Supplemental table S2** Patient characteristics of dengue fever (DF) cases with either primary or secondary infection.

|  | **Serolgical status** | |
| --- | --- | --- |
|  | **Primary** | **Secondary** |
| **Number of cases** | 11 | 16 |
| **Sex (M/F)** | 6/5 | 11/5 |
| **Age (mean(SD))** | 7.6 (3.3) | 8.9 (2.5) |
| **Dengue serotypes** |  |  |
| **DENV1** | 3 | 3 |
| **DENV2** | 4 | 5 |
| **DENV3** | 4 | 4 |
| **DENV4** | 0 | 4 |
| **Hemoconcentration (%)*(mean(SD))** | 15.1(5.1)* | 8.5 (8.1) |
| **Lowest platelet counts (mean(SD)** | 163909 (72308)* | 110563 (45659) |
| **Peak AST (u/dl) (mean(SD)** | 107 (94) | 111 (94) |
| **Peak ALT (u/dl) (mean(SD)** | 35 (41) | 56 (38) |
| **Lowest albumin (g/dl) (mean(SD)** | 4.3 (.35) | 4.09 (.53) |

* Indicates statistically significant difference between cases with a primary and a secondary infection by Student’s T test (P < .05)
