# Supplementary material for: Dengue Viral RNA Levels in Peripheral Blood Mononuclear Cells Are Associated with Disease Severity and Preexisting Dengue Immune Status
Source: PLoS One. 2012 Dec 19;7(12):e51335. doi: 10.1371/journal.pone.0051335 (PMC3526575; doi:10.1371/journal.pone.0051335)
Supplement: Table S3 — Primers used in positive and negative strand RT-PCR. (DOCX) [file pone.0051335.s003.docx]

**Supplementary table S3** Primers used in positive and negative strand RT-PCR

| Primers/Probes | Hybridization site | Used as | Nucleotide sequence |
| --- | --- | --- | --- |
| D1 forward primer | 10543-10566 | Forward primer in PCR of positive strand derived cDNA | 5’ ACA CCA GGG GAA GCT GTA TCC TGG 3’ |
| D2 forward primer | 10549-10572 | Forward primer in PCR of positive strand derived cDNA | 5’ AAG GTG AGA TGA AGC TGT AGT CTC3 ’ |
| D3 forward primer | 10523-10546 | Forward primer in PCR of positive strand derived cDNA | 5’ AGC ACT GAG GGA AGC TGT ACC TCC 3’ |
| D4 forward primer | 10476-10499 | Forward primer in PCR of positive strand derived cDNA | 5’ AAG CCA GGA GGA AGC TGT ACT CCT 3’ |
| D1,2,3 reverse primer | D1 10674-10694  D2 10680-10700  D3 10653-10673 | Primer in reverse transcription for positive strand RNA  Reverse primer in PCR | 5’ CAT TCC ATT TTC TGG CGT TCT 3’ |
| D4 reverse primer | 10605-10625 | Primer in reverse transcription for positive strand RNA  Reverse primer in PCR | 5’ CAA TCC ATC TTG CGG CGC TCT 3’ |
| TagF | D1 10571-10595  D2 10578-10602  D3 10552-10576  D4 10505-10529 | Primer in reverse transcription for negative strand RNA | 5’ CGG TCA TGG TGG CGA ATA A AA GGA CTA GAG GTT AKA GGA GAC CC 3’ |
| Tag |  | Forward primer in PCR for negative strand RNA derived cDNA | 5’ CGG TCA TGG TGG CGA ATA A 3’ |
| D1,2,3 probe | D1 10647-10672  D2 10653-10678  D3 10626-10651 | Probe for both positive and negative strand PCR | 5’ CTG TCT CCT CAG CAT CAT TCC AGG CA 3’ |
| D4 probe | 10578-10603 | Probe for both positive and negative strand PCR | 5’ CTG TCT CTG CAA CAT CAA TCC AGG CA 3’ |
