# Supplementary material for: Dengue Viral RNA Levels in Peripheral Blood Mononuclear Cells Are Associated with Disease Severity and Preexisting Dengue Immune Status
Source: PLoS One. 2012 Dec 19;7(12):e51335. doi: 10.1371/journal.pone.0051335 (PMC3526575; doi:10.1371/journal.pone.0051335)
Supplement: Table S4 — DENV RNA in C6/36 cells detected by positive and negative strand PCR. (DOCX) [file pone.0051335.s004.docx]

**Supplementary table S4** DENV RNA in C6/36 cells detected by Positive and negative strand PCR

| **Source of RNA** | **Positive strand PCR**  **(copies/ml)** | **Negative strand PCR**  **(copies/ml)** |
| --- | --- | --- |
| **DENV infected C6/36: cellular RNA** |  |  |
| **D2** | 7.65 X 10^7^ | 130868 |
| **D3** | 2.34 X 10^7^ | 487000 |
| **D4** | 5.54 X 10^6^ | 54638 |
| **Tag primer omitted during PCR step** | Not done | undetected |
| **Uninfected C6/36: cellular RNA** | undetected | undetected |
